# Neurostimulation device infection control in China and the United States: a comparative analysis using the MCS framework

**DOI:** 10.3389/fneur.2025.1755822

**Published:** 2026-02-09

**Authors:** Xiaohan Qin, Lanfang Chen

**Affiliations:** Central Sterile Supply Department, Wuxi Hospital of Traditional Chinese Medicine, Wuxi, China

**Keywords:** antimicrobial resistance, China, deep brain stimulation, healthcare policy, infection control, neurostimulation, spinal cord stimulation, United States

## Abstract

Neurostimulation devices, including deep brain stimulation (DBS) and spinal cord stimulation (SCS) systems, have transformed treatment for neurological disorders and chronic pain. However, device-related infections remain a critical challenge with global incidence rates of 3–7%. This narrative review introduces the Mechanism-Clinical-System (MCS) framework to comprehensively evaluate infection control practices in China and the United States, integrating evidence from 2020 to 2025. At the mechanism level, fundamental differences in sterilization technologies—ethylene oxide dominance in the US versus hydrogen peroxide plasma preference in China—create distinct operational profiles, though clinical outcomes appear equivalent. China’s 10-fold higher antibiotic consumption drives elevated antimicrobial resistance (MRSA: 60–75% vs. 40–55%). Clinically, DBS infection rates remain comparable between countries (US: 3.5–6.5%; China: 5.7%), while prolonged antibiotic prophylaxis (5–14 days) persists in China despite evidence supporting 24-h protocols. At the system level, divergent regulatory frameworks—FDA mandatory compliance versus NMPA’s tiered implementation—create fundamental practice variability. Neither healthcare system demonstrates uniform superiority. The US achieves greater standardization through regulatory stringency, while China demonstrates remarkable adaptability and innovation velocity. Evidence-based harmonization strategies—including international registries, standardized surveillance, and antimicrobial stewardship—offer substantial potential to optimize patient safety globally.

## 1 Introduction

### 1.1 Global burden of neurostimulation device infections

The exponential growth of neurostimulation therapies has transformed management of treatment-resistant neurological and pain disorders. Deep brain stimulation (DBS), now standard-of-care for advanced Parkinson’s disease, essential tremor, and dystonia, has expanded to novel indications including treatment-resistant depression and obsessive-compulsive disorder (1, 2). The global DBS market reached USD 1.40 billion in 2024, with projections of USD 2.50 billion by 2030 (3). Similarly, spinal cord stimulation (SCS) has shown remarkable efficacy for complex regional pain syndrome and failed back surgery syndrome, with over 50,000 annual implantations globally (4, 5).

Despite technological advances, device-related infections remain a persistent challenge. A comprehensive meta-analysis analyzing 15,842 DBS procedures reported pooled infection rates of 5.0% (95% CI: 4.4–5.6%), with significant variation by indication: epilepsy (9.5%), dystonia (6.5%), and Parkinson’s disease (4.2%) (6). The JAMA Neurology INSITE registry documented SCS infection incidence of 2.8% (95% CI: 2.3–3.4%), with higher rates in revision procedures (4.1%) versus primary implantations (2.3%) (7).

### 1.2 The MCS framework

Traditional analyses of medical device infections have focused on isolated domains—mechanistic studies, clinical trials, or policy evaluations. This fragmented approach fails to capture the complex interplay between biological, clinical, and systemic factors. We propose the Mechanism-Clinical-System (MCS) framework as an innovative analytical tool integrating these perspectives.

The Mechanism axis encompasses microbial pathogenesis, biofilm dynamics, sterilization technologies, and antimicrobial resistance. The Clinical axis addresses infection epidemiology, risk stratification, surgical techniques, and prophylactic protocols. The System axis examines regulatory frameworks, payment models, quality monitoring, and organizational culture.

### 1.3 Rationale for Sino-US comparative analysis

China and the United States represent the world’s two largest healthcare markets with fundamentally different structures (8). The US performs approximately 12,000 DBS procedures annually across 150 centers, while China’s 200 + centers demonstrate greater volume heterogeneity (10–300 + cases/year) (9, 10). These structural differences create distinct infection control challenges and opportunities for bidirectional learning.

## 2 Mechanism axis

### 2.1 Device architecture and material constraints

Contemporary neurostimulation systems share architectural features creating inherent sterilization challenges. DBS systems comprise intracranial electrodes (platinum-iridium contacts insulated with polyurethane or silicone), extension cables (40–60 cm), and implantable pulse generators (IPGs) in titanium enclosures (2, 11). The dominant manufacturers include Medtronic (Ireland/USA), Abbott (USA), and Boston Scientific (USA), which together account for >85% of the global market. In China, PINS Medical Technology (Beijing) and SceneRay (Suzhou) have emerged as domestic manufacturers with increasing market share (11). SCS systems introduce additional complexity through epidural lead designs with extensions measuring 25–100 cm (13).

The fundamental sterilization constraint derives from thermal sensitivity: internal electronics tolerate maximum 50–60 °C, while batteries risk thermal runaway above 65 °C (14). Steam autoclaving (121–134 °C) is therefore contraindicated. Polyurethane insulation undergoes hydrolytic degradation with repeated moisture-heat exposure (15).

Hardware infections demonstrate predictable anatomical patterns: IPG pocket (44%), lead/extension components (34%), with multi-site involvement in 22% (16). *Staphylococcus epidermidis* and *S. aureus*, implicated in >60% of infections, elaborate biofilm matrices conferring 100–1,000-fold increased antibiotic resistance—frequently necessitating hardware explantation (17, 18).

### 2.2 Sterilization modalities

Ethylene oxide (EtO) remains predominant in US facilities, achieving microbicidal effects through irreversible alkylation (19). Cycles operate at 37–63 °C over 1–6 h, followed by 12–16 h aeration (20). EtO offers exceptional penetration but requires sophisticated ventilation for OSHA compliance and extended cycle times exceeding 12–18 h (21, 22).

Hydrogen peroxide (H₂O₂) plasma sterilization has achieved increasing prevalence in China. Systems employ H₂O₂ vapor at 37–50 °C with radiofrequency-generated plasma (11, 24). Cycles complete within 45–75 min with no environmental hazards (25). The principal limitation involves reduced penetration through extended lumens, though current-generation systems demonstrate improved capability (26, 27).

**Figure 1:**
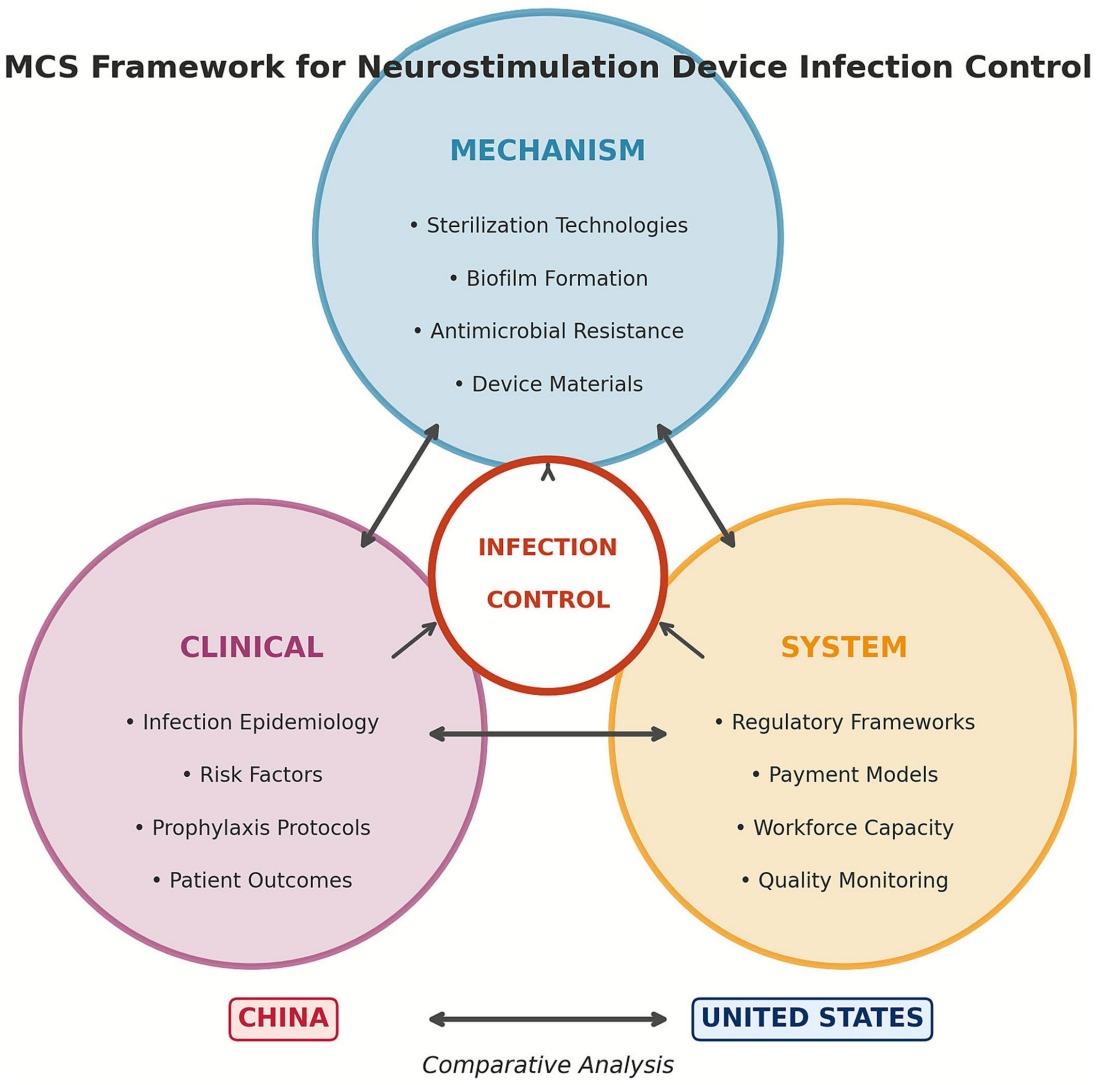
The Mechanism-Clinical-System (MCS) Framework for analyzing neurostimulation device infection control. The framework integrates three interconnected axes: Mechanism (microbial pathogenesis, sterilization technologies, antimicrobial resistance), Clinical (infection epidemiology, prophylaxis protocols, surgical techniques), and System (regulatory frameworks, payment models, workforce training).

Clinical outcomes appear equivalent between modalities. US centers report DBS infection rates of 3.5–6.5% (28), comparable to European plasma-utilizing centers (3.8–6.2%) (29). Chinese facilities transitioning to plasma report reductions from 7–9% to 5–7% (30) (Figure 1).

Table 1 Comparison of ethylene oxide (EtO) and hydrogen peroxide plasma sterilization technologies for neurostimulation devices. Parameters include cycle time, temperature range, penetration capability, environmental considerations, and clinical infection outcomes.

**Table 1:** Comparison of low-temperature sterilization modalities for neurostimulation devices.

Parameter	Ethylene oxide (EtO)	H₂O₂ plasma
Predominant Region	United States	China
Temperature	37–63 °C	37–50 °C
Cycle Duration	1–6 h + 12–16 h aeration	45–75 min
Total Turnaround	12–18 h	<2 h
Penetration Capability	Excellent (complex geometries)	Moderate (improved in newer systems)
Material Compatibility	Excellent	Good (cellulose contraindicated)
Environmental Concerns	Carcinogenic; OSHA regulated	None (H₂O + O₂ byproducts)
Capital Cost	$50,000–500,000	$80,000–200,000
Operating Cost/Cycle	$25–40	$50–100
Clinical Infection Rates	3.5–6.5%	3.8–6.2%*

*European and Chinese data using predominantly plasma sterilization. EtO, ethylene oxide; H₂O₂, hydrogen peroxide; OSHA, Occupational Safety and Health Administration.

### 2.3 Antimicrobial resistance patterns

Resistance profiles differ substantially between countries. MRSA prevalence averages 40–55% in the US versus 60–75% in China (31, 32), reflecting antibiotic consumption patterns: China’s per-capita use (~138 DDD/1000/day) exceeds the US (~13.6 DDD/1000/day) by 10-fold (33). Coagulase-negative staphylococci demonstrate methicillin resistance exceeding 70–85% in Chinese isolates versus 40–60% in the US (34).

These patterns directly influence prophylaxis strategies. US practice regarding routine MRSA prophylaxis remains controversial, while Chinese centers increasingly employ combination regimens despite limited supporting evidence (35, 36).

### 2.4 Antimicrobial prophylaxis divergence

US standard practice employs weight-based cefazolin (2–3 g) administered 30–60 min pre-incision, limited to 24 h (37, 38). Chinese practice demonstrates greater heterogeneity: median prophylaxis of 5–7 days, with 30–40% of centers continuing 10–14 days (39, 40). Multiple factors drive extended prophylaxis: higher MRSA prevalence, medicolegal pressures, and longer operative times (4–6 h vs. 3–4 h) (41).

Evidence strongly supports short-duration protocols. Meta-analysis confirmed no benefit from prophylaxis exceeding 24 h (OR 0.98, 95% CI 0.63–1.52) while documenting increased resistance (42, 43). Chinese investigators reported infection reductions from 7.9 to 5.3% after shortening prophylaxis from 7 to 3 days (44).

## 3 Clinical axis

### 3.1 Infection epidemiology

Meta-analysis of 66 studies (12,258 patients) reported pooled DBS surgical site infection prevalence of 5.0% (95% CI: 4.0–6.0%), with considerable heterogeneity by indication (6). Infection timing demonstrates trimodal distribution: early (<30 days, 35%), intermediate (30–90 days, 40%), and late (>90 days, 25%) (16). Early infections typically reflect perioperative contamination, while late infections suggest hematogenous seeding or delayed-onset biofilm maturation (45).

**Figure 2:**
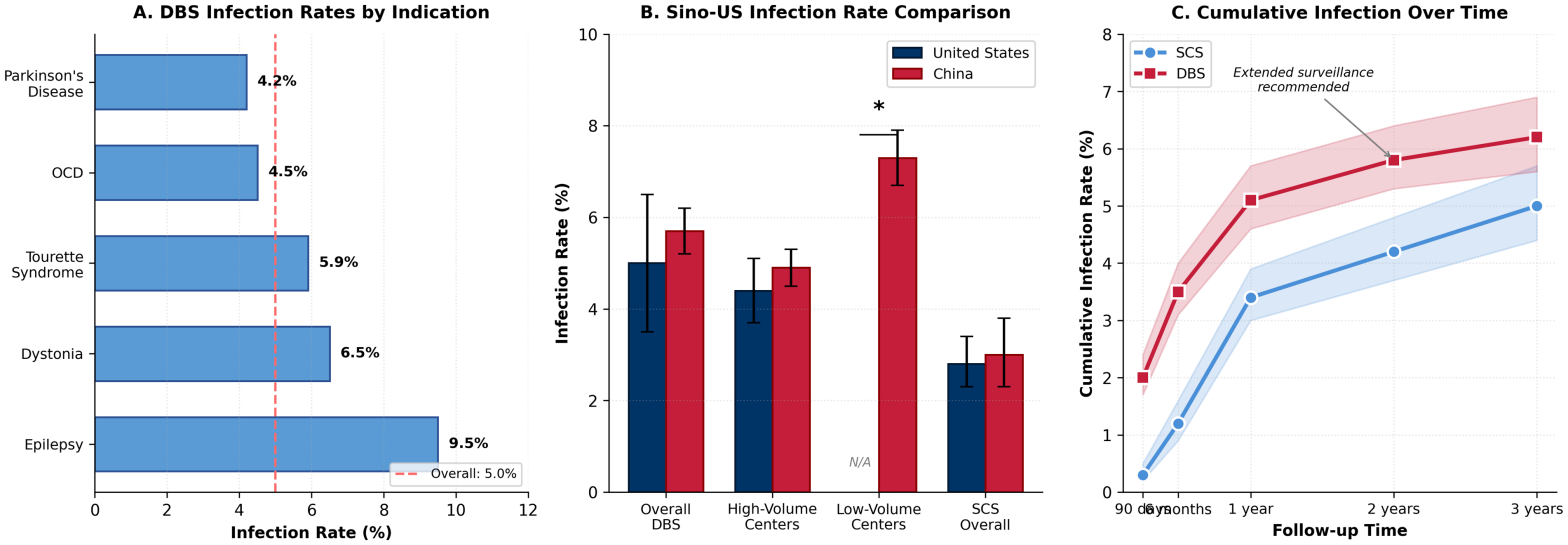
Comparison of DBS and SCS infection rates between China and the United States. **(A)** Overall infection rates by device type; **(B)** Infection rates by procedure volume; **(C)** Temporal distribution of infections (early, intermediate, late).

Chinese registry data (1,250 procedures) reported 5.7% infection rate, with significant volume-outcome relationships: centers performing >50 annual procedures demonstrated 4.9% versus 7.3% in lower-volume facilities (10). Robot-assisted DBS procedures show promise for reducing operative time and potentially infection risk (41) (Figure 2).

### 3.2 Risk stratification and prevention

Established risk factors include diabetes mellitus, immunosuppression, revision surgery, prolonged operative time, and previous surgical site infections (46, 47). Machine learning models incorporating preoperative variables demonstrate promising predictive accuracy (AUC 0.78–0.85) for identifying high-risk patients (48, 49).

Preventive interventions with strong evidence include chlorhexidine-alcohol skin preparation, antibiotic-impregnated envelopes for IPG placement, and standardized surgical bundles (50, 51). Novel approaches under investigation include antimicrobial coatings, bacteriophage therapy, and immunomodulation strategies (52, 53).

## 4 System axis

### 4.1 Regulatory frameworks

The US regulatory system operates through layered authorities. FDA mandates sterility assurance levels of 10^−6^, with facilities legally obligated to follow manufacturer Instructions for Use (21). CMS enforces infection control through Conditions of Participation affecting 40–60% of hospital revenue (54). The NHSN enables standardized surveillance across >6,800 facilities (55), while the Hospital-Acquired Condition Reduction Program penalizes worst-performing quartile institutions (56).

China’s NMPA provides primary regulatory authority, with recent reforms reducing Class III approval times from 3–4 years to 18–24 months (57, 58). The WS 310 standards specify CSSD requirements, though enforcement varies by facility tier (59). The China Hospital Infection Control Network encompasses approximately 2,500 hospitals (20% of eligible facilities), with surveillance remaining largely voluntary (60).

Table 2 Comparison of regulatory frameworks for neurostimulation device infection control between China and the United States. Categories include regulatory authority, sterility standards, surveillance systems, and quality incentive programs.

**Table 2:** Comparison of antimicrobial prophylaxis protocols for neurostimulation surgery.

Parameter	United States	China
First-line Agent	Cefazolin 2–3 g	Third-generation cephalosporins (40–50%)
Timing	30–60 min pre-incision	Variable
Duration	≤24 h	5–7 days (median); 30–40% continue 10–14 days
MRSA Coverage	Controversial; added in high-prevalence settings	Combination regimens in 25–35% of centers
Alternative Agents	Vancomycin, clindamycin	Vancomycin + cephalosporin combinations
Guideline Adherence	High (IDSA/ASHP)	Variable; national stewardship since 2011
MRSA Prevalence	40–55%	60–75%
Antibiotic Consumption	13.6 DDD/1000/day	138 DDD/1000/day

DDD, defined daily doses; IDSA, Infectious Diseases Society of America; ASHP, American Society of Health-System Pharmacists; MRSA, methicillin-resistant *Staphylococcus aureus*.

### 4.2 Healthcare financing

DBS implantation receives Medicare DRG reimbursement averaging $35,000–45,000, with surgical site infections generating $30,000–50,000 in uncompensated costs (54, 61). Value-based purchasing ties 3–4% of payments to quality metrics (62). Healthcare-associated infections cost the US healthcare system an estimated $28–45 billion annually (54).

China’s DRG-like systems (DIP) cover 80% of major-city admissions since 2017–2019 (63). Neurostimulation receives bundled payments of USD 11,000–17,000 (¥80,000–120,000), with infections not qualifying for additional payment (40). However, quality-based adjustments remain minimal (<1% vs. 3–4% in US) (56) (Table 3).

**Table 3:** Comparative analysis of healthcare system infrastructure for neurostimulation infection control.

Domain	United States	China
Regulatory framework		
Primary authority	FDA (21 CFR Part 820)	NMPA
Enforcement	Mandatory; legal liability	Tiered; variable by facility level
Approval timeline	Established pathways	Reduced: 3–4 years → 18–24 months
Surveillance system		
National network	NHSN (>6,800 facilities)	CHIC Network (~2,500 facilities)
Participation	Mandatory (most states)	Largely voluntary
Risk adjustment	Sophisticated	Limited
Payment model		
Reimbursement	DRG ($35,000–45,000)	DIP (¥80,000–120,000)
Quality adjustment	3–4% of payments	<1% of payments
Infection cost	Hospital absorbs	Hospital + patient (30–50% OOP)
Workforce capacity		
CSSD certification	Formal (400 h + exam)	Variable (12–68% by tier)
IP ratio	1:100–150 beds	1:200–250 beds
Continuing education	12–15 credits/year required	Variable requirements
Infrastructure		
OR air quality	ANSI/ASHRAE 170 (≥20 ACH)	WS 310 (variable compliance)
Instrument tracking	Electronic (barcode/RFID)	Paper to electronic (tier-dependent)
Safety culture	Flat hierarchy; sterile conscience	Evolving; traditional hierarchy persists

FDA, Food and Drug Administration; NMPA, National Medical Products Administration; NHSN, National Healthcare Safety Network; CHIC, China Hospital Infection Control; DRG, diagnosis-related group; DIP, Diagnosis-Intervention Packet; OOP, out-of-pocket; CSSD, Central Sterile Supply Department; IP, infection preventionist; OR, operating room; ACH, air changes per hour; RFID, radio-frequency identification.

### 4.3 Workforce and infrastructure

US CSSD technicians complete formal certification (400 h plus examination) with annual continuing education (59, 64). Chinese certification rates vary substantially: 68% in Beijing/Shanghai tier-3 hospitals versus 12% in tier-1 facilities (30, 60). US hospitals feature ≥1 infection preventionist per 100–150 beds versus 1 per 200–250 beds in China (44, 65). Leading Chinese centers feature world-class environments, though mid-tier hospitals rely on manual cleaning and paper-based tracking (60, 66).

## 5 Discussion

### 5.1 Synthesis of findings

The MCS framework reveals synergistic interactions across dimensions. Mechanism-level constraints (thermal sensitivity, biofilm dynamics) create universal challenges, while system factors determine technology choices. Antimicrobial resistance shaped by consumption policies necessitates different prophylaxis strategies. Clinical infection rates remain elevated despite decades of experience, with practice patterns reflecting system constraints including workforce gaps and economic pressures. Neither regulatory model optimally balances standardization, innovation, and equity.

### 5.2 Drivers of practice divergence

US infection control evolved incrementally over 50 + years, creating embedded investment in specific technologies (67). China’s healthcare modernization compressed decades into 15–20 years, enabling technology leapfrogging but challenging infrastructure development (8). US per-capita health expenditure ($12,555) exceeds China’s ($936) by 13.4-fold, though Chinese hospitals benefit from bulk purchasing discounts and lower labor costs (8, 56).

Cultural factors influence implementation: US medical culture emphasizes litigation avoidance generating strong protocol adherence incentives, while Chinese operating rooms are evolving toward flat hierarchy models following WHO Surgical Safety Checklist adoption (59, 68).

### 5.3 Opportunities for mutual learning

US practices applicable to China: Expanding mandatory infection surveillance from 20% to ≥80% facility participation; increasing quality-based reimbursement adjustments from <1% to 3–5%; implementing nationally recognized professional certifications.

Chinese innovations applicable to US: Tiered implementation models accommodating facility heterogeneity; streamlined regulatory pathways accelerating innovation access; comprehensive digital health integration for workflow optimization (66, 69).

**Figure 3:**
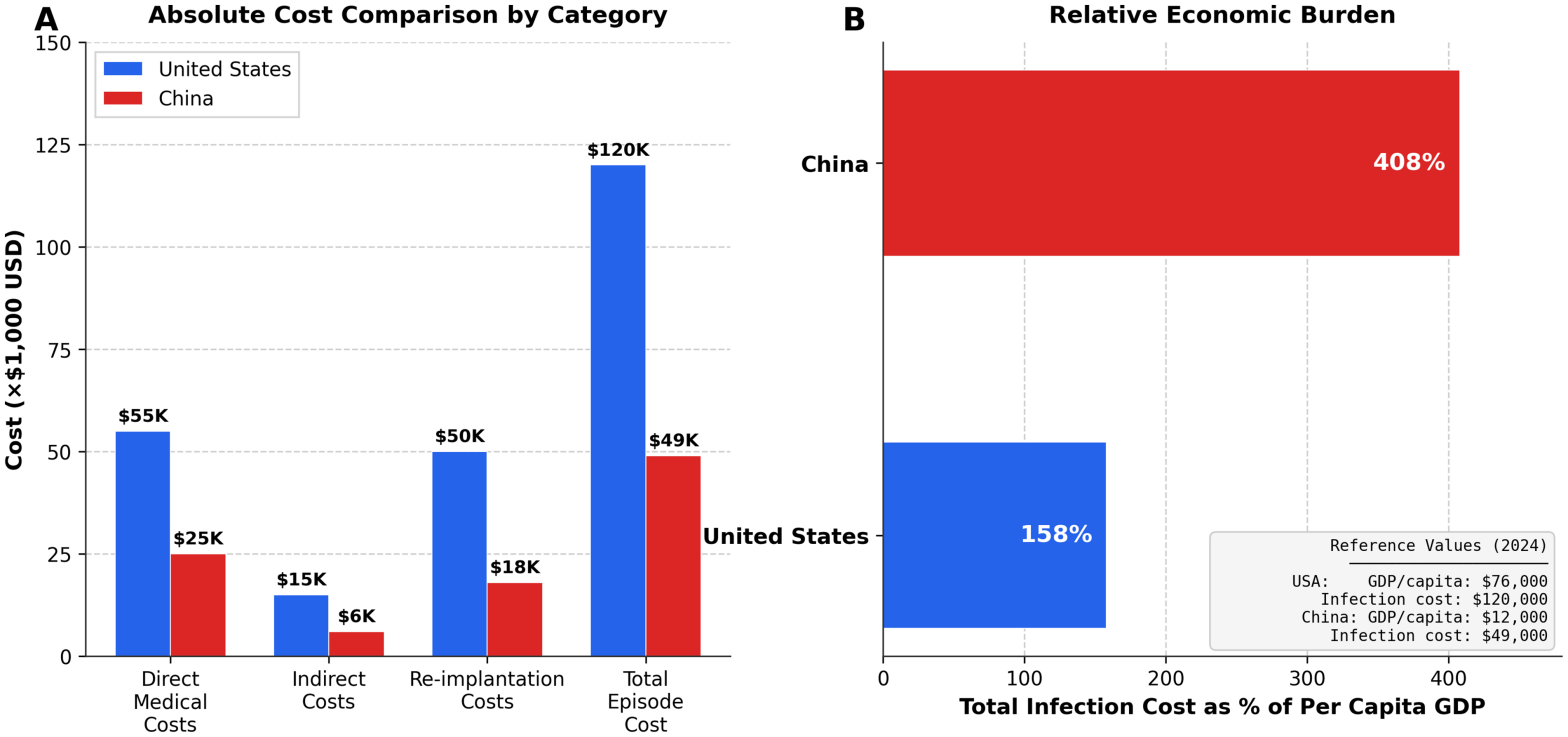
Bidirectional learning opportunities between China and the United States for neurostimulation device infection control. Arrows indicate knowledge transfer directions, with key practices and innovations highlighted for each country.

System-level implementation considerations: Successful adoption of these recommendations requires addressing several practical barriers. For surveillance system expansion in China, phased implementation beginning with provincial centers of excellence, supported by standardized electronic reporting templates and dedicated infection control personnel, could achieve 50% coverage within 3–5 years. Quality-based reimbursement adjustments necessitate development of validated, risk-adjusted outcome metrics that account for case complexity and patient comorbidities. Professional certification programs require investment in training infrastructure, examination development, and continuing education platforms. Cross-national knowledge transfer may be facilitated through joint professional society initiatives, international fellowship exchanges, and collaborative research networks building on existing relationships between academic medical centers (Figure 3).

### 5.4 Limitations

This narrative review did not employ exhaustive search strategies or meta-analytic methods. Data quality varies substantially—US surveillance benefits from mature NHSN infrastructure while Chinese data derives from more limited networks. Specifically, Chinese infection surveillance data may underestimate true incidence due to voluntary reporting mechanisms, variable case definitions across institutions, and potential underreporting incentives within performance-based assessment systems. Additionally, published Chinese studies predominantly originate from tier-3 academic medical centers in major cities (Beijing, Shanghai, Guangzhou), limiting generalizability to the broader healthcare system including tier-1 and tier-2 facilities serving rural populations. Direct Sino-US comparisons derive from separate cohorts rather than designed comparative research. The MCS framework requires empirical validation; while the framework provides a useful conceptual structure for organizing complex multi-level factors, prospective studies are needed to determine whether MCS-guided interventions yield superior outcomes compared to conventional approaches. Future validation efforts should include multi-center trials testing framework-derived hypotheses and comparative effectiveness research across diverse healthcare settings.

### 5.5 Future directions

Priority research includes comparative sterilization studies with clinical infection outcomes, pragmatic trials comparing prophylaxis duration, and machine learning-based risk prediction models (48, 70). System-level initiatives should prioritize international registries operated by professional societies, health economic modeling comparing regulatory approaches, and implementation science addressing the evidence-practice gap (71, 72).

## 6 Conclusion

This review, employing the MCS framework, provides insights into neurostimulation device infection control across two distinct healthcare systems. Neither demonstrates uniform superiority: the US achieves greater standardization through regulatory stringency, while China demonstrates remarkable innovation velocity and adaptability.

Practical implications include evidence-based guidance on prophylaxis duration (24 h), antiseptic selection (chlorhexidine-alcohol), and risk stratification. Administrators should prioritize surveillance systems, stewardship programs, and value-based payment models. Policymakers must balance standardization with flexibility.

Eliminating preventable infections requires systems thinking and sustained commitment. International collaboration through shared registries and comparative effectiveness research will accelerate progress beyond what either country could achieve independently. The MCS framework illuminates the path forward: integrated interventions spanning basic science, clinical practice, and health policy.
